# Treatment delay of bone tumours, compilation of a sociodemographic risk profile: A retrospective study

**DOI:** 10.1186/1471-2407-8-22

**Published:** 2008-01-23

**Authors:** Christoph Schnurr, Mathias Pippan, Hartmut Stuetzer, Karl S Delank, Joern WP Michael, Peer Eysel

**Affiliations:** 1 Department of Orthopedic Surgery, University of Cologne, Joseph-Stelzmann-Str.9, 50924 Cologne, Germany; 2 Sankt Josefs-Hospital, Solmsstraße 15, 65189 Wiesbaden, Germany; 3 Institute for Medical Statistics, Informatics and Epidemiology, University of Cologne, Kerpener Straße 62, 50937 Cologne, Germany

## Abstract

**Background:**

Bone tumours are comparatively rare tumours and delays in diagnosis and treatment are common. The purpose of this study was to analyse sociodemographic risk factors for bone tumour patients in order to identify those at risk of prolonged patients delay (time span from first symptoms to consultation), professional delay (from consultation to treatment) or symptom interval (from first symptoms to treatment). Understanding these relationships might enable us to shorten time to diagnosis and therapy.

**Methods:**

We carried out a retrospective analysis of 265 patients with bone tumours documenting sociodemographic factors, patient delay, professional delay and symptom interval. A multivariate explorative Cox model was performed for each delay.

**Results:**

Female gender was associated with a prolonged patient delay. Age under 30 years and rural living predisposes to a prolonged professional delay and symptom interval.

**Conclusion:**

Early diagnosis and prompt treatment are required for successful management of most bone tumour patients. We succeeded in identifying the histology independent risk factors of age under 30 years and rural habitation for treatment delay in bone tumour patients. Knowing about the existence of these risk groups age under 30 years and female gender could help the physician to diagnose bone tumours earlier. The causes for the treatment delays of patients living in a rural area have to be investigated further. If the delay initiates in the lower education of rural general physicians, further training about bone tumours might advance early detection. Hence the outcome of patients with bone tumours could be improved.

## Background

Bone tumours are comparatively rare tumours; a report of the American Cancer Society revealed an estimate of 2500 new bone tumours annually for the whole of the United States [1]. Furthermore, symptoms of bone neoplasms are often vague and misleading [2]. Therefore, delays of diagnosis and treatment of these tumours are common [2-10]. There has been a remarkable increase in survival rates for various bone tumours in recent decades [11-14]. Therefore, a shortening of diagnostic and treatment delay must be an objective of successful bone tumour management [2,11,12,15,16]. Many studies have been undertaken to investigate treatment or prognosis of bone tumours, but studies about diagnostic and treatment delays are rare.

The period of time between first symptoms and initial treatment can be divided into two main categories: patient delay, which is defined as the time that passes between first symptoms and first consultation of a physician, and professional delay, which is mainly caused by the doctor and defined as the time span between first consultation and initial treatment. The addition of these two delays, the time from first symptoms to the first treatment, is called overall symptom interval [6].

The goal of shortening these delays might be attained by identifying the "at risk" group of patients through sociodemographic risk factors. These factors might be attributed to the patient, the doctor or both and are broadly known for many other tumour types [17-20].

Delays of diagnosis for bone tumours have been studied for children [5,6,19] and to some extent for older patients [2,3]. But in these studies only a specific tumour type was analysed and therefore the identified risk factors are only tumour type specific. It remains unclear if some "more universal" risk factors for diagnostic and treatment delays of bone tumour patients beneath tumour type specific ones exist.

However so far to our knowledge no study has been undertaken aiming at developing such a non tumour type specific sociodemographic risk profile.

Therefore, the purpose of our study was the analysis of sociodemographic risk factors in order to identify the endangered patient for a prolonged diagnostic or treatment delay. Understanding these relationships might enable us to improve diagnosis and treatment of bone tumour patients.

## Methods

We carried out a retrospective analysis at the senior author's clinic from 1988 to 1998. Patients with a primary or secondary bone tumour that meet one of the following criteria have been included into our study (n = 265): a) tumours differentiation could not be clarified in the X-ray examination and a diagnostic surgical biopsy was necessary b) benign tumours that required surgical filling due to biomechanical reasons to prevent fractures.

To develop a sociodemographic risk profile, the following data were assembled: date of birth, gender, profession (scholars, employees, non-employed persons, pensioners), place of residence [village/small town (<50000), metropolis (>50000)], kind of habitation (apartment sharing, single), nationality (native, foreign).

The histology of the tumour was recorded and grouped according to the differentiation of the tumour (benign, semi benign and malignant) and genesis (primary bone tumour, metastases with known primary tumour, metastases with unknown primary tumour).

For each patient three time-points had been recorded: date of first symptoms, date of first consultation of a medical doctor, date of first specific treatment. A patient was considered to be symptomatic from the day that unrelieved symptoms, directly attributable to the bone tumour, were first recorded. As a first specific treatment, operative and non operative procedures such as chemotherapy or conservative therapy by a surgical corset were defined. The following periods of time were calculated by use of these three dates [6]: 1. Patient delay: time from first occurrence of symptoms of the tumour to the first medical consultation. 2. Professional delay: time from the first medical consultation to the first specific treatment. 3. Symptom interval: total period between presenting first symptoms and the first specific treatment.

### Statistics

All time to event data were analysed for significant associations with explanatory factors on the length of time intervals using nonparametric methods to avoid special assumptions on the distributions of the underlying data: Kaplan-Meier estimates of cumulated rates when analysing univariant risks and Cox proportional hazards regression models to fit multivariate risk patterns using backward elimination and forward selection algorithms based on likelihood ratio statistics. Hazard ratios (HR) are given together with their 95% confidence intervals (95%-CI) as resulting from the final model step when using stepwise algorithms or intermediate steps as indicated. Due to the modelling of the length of the delay intervals, hazard ratios lower than 1 correspond to prolonged delay intervals and hazard ratios higher than 1 correspond to shortened ones. Reflecting the hypotheses generating character of our analyses, all p-values cited are given as nominal values, i.e. they are uncorrected for multiple testing situations and consequently may be interpreted merely in an explorative manner. All computations were done using SPSS^® ^for Windows^® ^software (version 12).

## Results

A summary of the investigated sociodemographic factors is shown in Table 1. The age of the 265 patients at presenting first symptoms was in mean 50 years (range 5 to 87 years).

**Table 1:** Sociodemographic factors. Summary of the acquired sociodemographic factors of the investigated population. Additionally, genesis and differentiation of the tumour are listed.

	**Number (n)**	**Percent**
**Patients**	265	100
**Gender**		
female	143	54.0
male	122	46.0
**Age**		
0–30	51	19.2
30–60	114	43.0
60–90	100	37.7
**Profession**		
employees	88	33.2
scholars	28	10.6
non-employed persons	40	15.1
pensioners	109	41.1
**Place of residence**		
villages/small towns	161	60.8
metropolis	104	39.2
**Kind of habitation**		
apartment sharing	202	76.2
single	63	23.8
**Nationality**		
native	251	94.7
foreign	14	5.3
**Differentiation**		
benign	80	30.2
semi malignant	20	7.5
malignant	165	62.3
**Genesis**		
primary bone tumour	140	52.8
metastases without known primary tumour	50	18.9
metastases with known primary tumour	75	28.3

**Figure 1:**
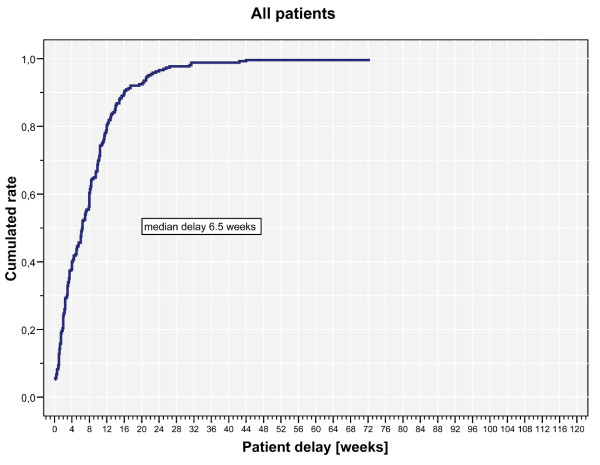
**Kaplan Meier chart of patient delay.** A mean patient delay of 8 weeks (median 6.5 weeks; range 0 to 72.1 weeks). The number 0 was given when a patient never had symptoms attributed to a tumour before diagnosis.

**Figure 2:**
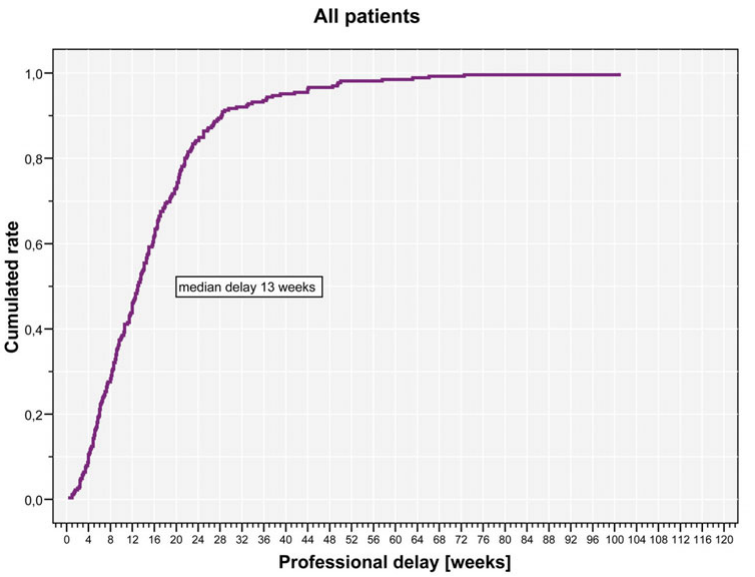
**Kaplan Meier chart of professional delay.** A diagrammed a mean professional delay of 15.7 weeks (median 13 weeks; range 0.3 to 100.9 weeks).

**Figure 3:**
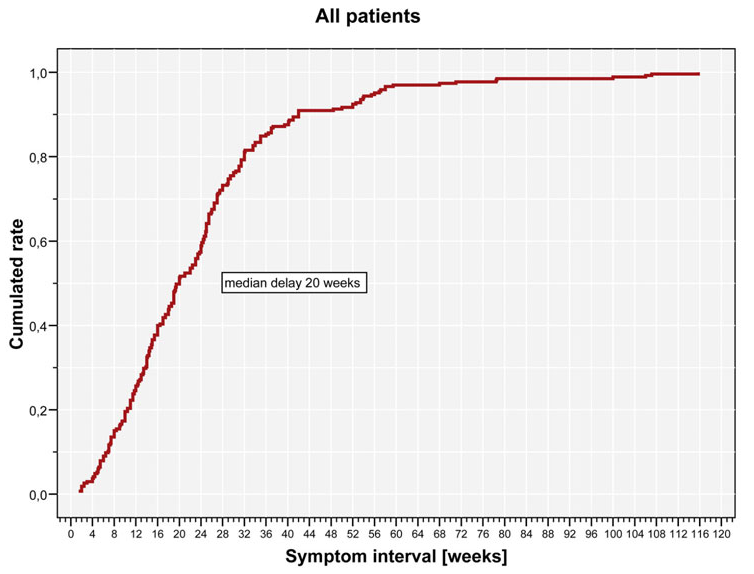
**Kaplan Meier chart of symptom interval.** The summary of the delays, the so-called symptom interval, is shown. The mean symptom interval was 23.5 weeks (median 20 weeks; range 1.4 to 115.7 weeks).

Histological analyses of the tumours yielded 40 malignant primary bone tumours, 80 benign bone tumours, 20 semi malignant bone tumours and 125 metastases (Table 2). Analysing the patient delay, we calculated a mean patient delay of 8 weeks (range 0 to 72.1 weeks, Figure 1). The mean professional delay was 15.7 weeks (range 0.3 to 100.9 weeks, Figure 2), the mean symptom interval 23.5 weeks (range 1.4 to 115.7 weeks, Figure 3). A summary of the delays attributed to sociodemographic criteria is shown in Table 3.

**Table 2:** Histology. Histology of the 265 tumours is listed below.

**Histology**	**Number (n)**	**Percent**
Chondro-/Osteosarcoma	40	15.1
Giant cell tumour/aggressive fibromatosis	20	7.5
Osteoidosteoma	13	4.9
Other benign primary bone tumours	67	25.3
Metastases with known primary tumour	75	28.3
Metastases without known primary tumour	50	18.9
**Total**	**265**	**100**

**Table 3:** Summary of the delays for each sociodemographic factor. The detected patient delay, professional delay and symptom interval are shown for each sociodemographic factor. Furthermore, the delays for the subgroups genesis and differentiation of the tumour are listed.

**Patients**	**Patient delay (weeks)**	**Professional delay (weeks)**	**Symptom interval (weeks)**
**Gender**			
female	8.7	15.1	23.8
male	7	16.3	23.3
**Profession**			
employees	8.1	17.6	25.7
scholars	11.4	18.2	29.5
non-employed persons	12.7	11.8	24.4
pensioners	6.9	13.2	20.1
**Place of residence**			
villages/small towns	9.1	17.3	26.4
metropolis	6.3	12.9	19.2
**Kind of habitation**			
apartment sharing	8	15.9	23.9
single	7.7	14.7	22.4
**Nationality**			
native	7.9	15.7	23.6
foreign	8.8	14.6	23.4
**Differentiation**			
benign	10.8	20.5	31.3
semi malignant	10.1	19.2	29.3
malignant	6.2	12.9	19.1
**Genesis**			
primary bone tumour	10.5	19	29.5
metastases without known primary tumour	8.6	14.6	23.2
metastases with known primary tumour	2.7	10	12.7

### Cox Regression Model

**Figure 4:**
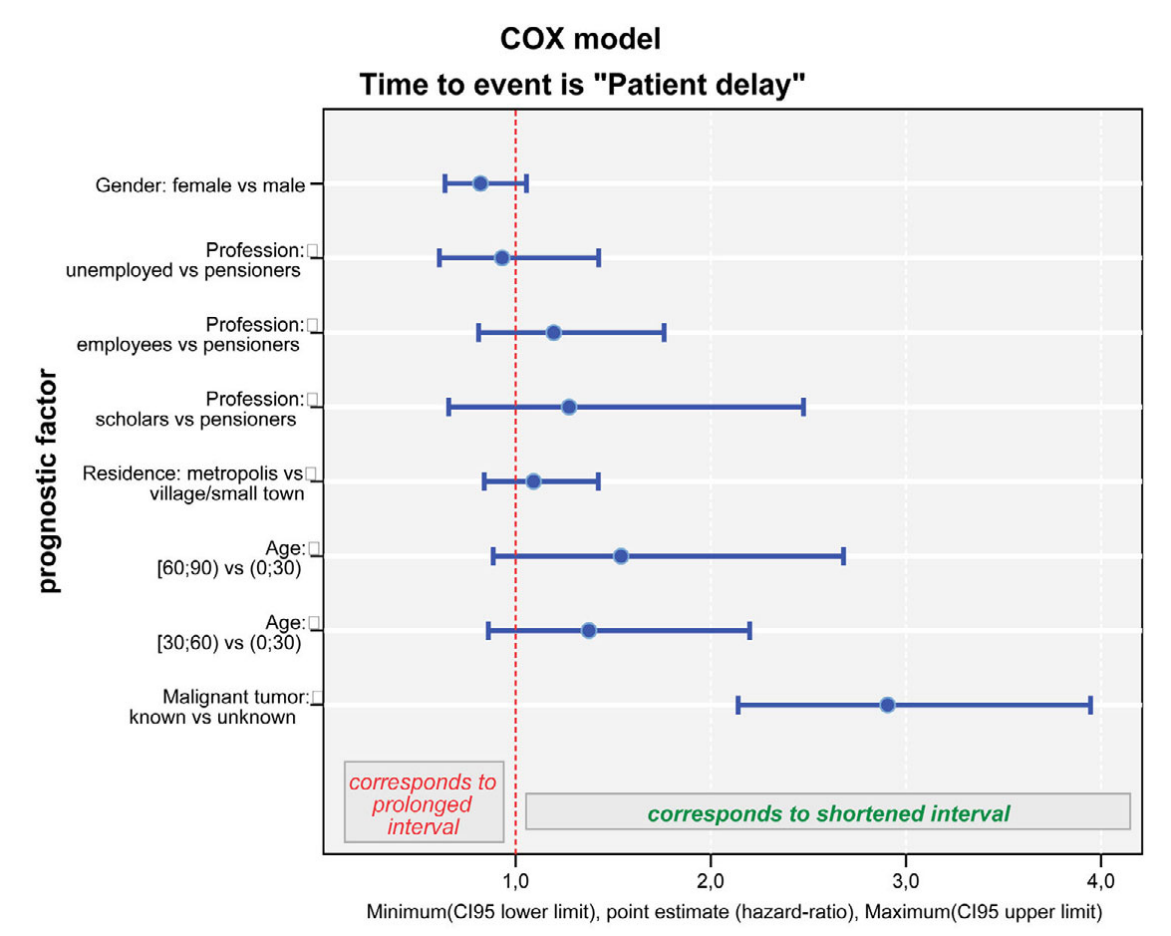
**Cox model of patient delay**. This diagram shows step 4 of the Cox model for patient delay. In these Cox diagrams blue bars on the left site of the pointed red line indicate a prolonged delay, the blue bars on the right site of the red line indicate a shortened delay. As diagrammed above, the criterion metastases of a known primary malignant tumour caused a significantly shorter patient delay in comparison to metastases of an unknown primary tumour or a primary bone tumour (p < 0.001). Female gender caused a prolongated patient delay (p = 0.072).

**Figure 5:**
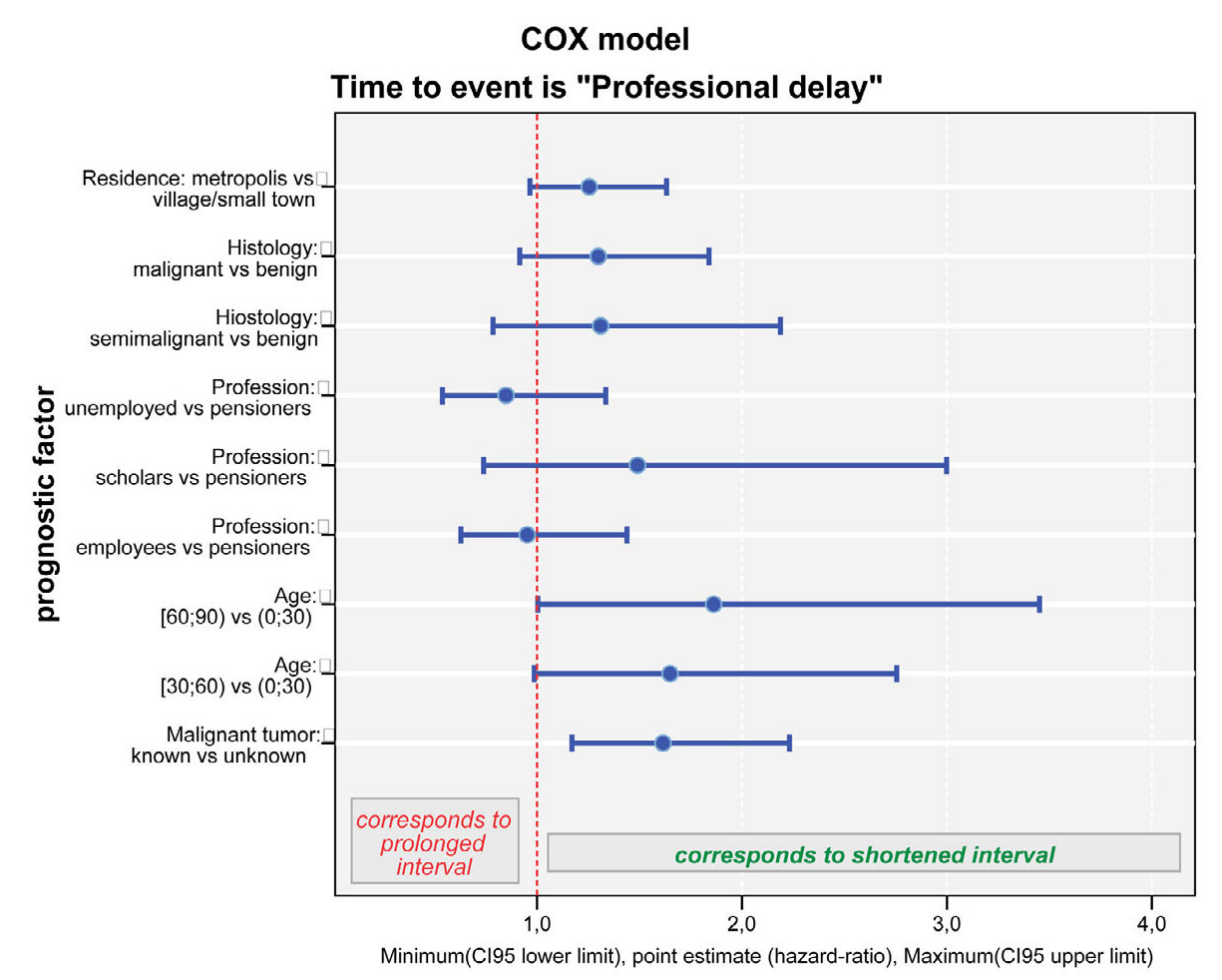
**Cox model of professional delay**. Intermediate step 4 of the final Cox model for professional delay demonstrates that the factor age <30 years had a significantly negative impact yielding a prolongation of the professional delay in comparison to elder patients (p = 0.007). Residence in a rural area predisposes to a prolonged professional delay (p = 0,097). The criterion known primary tumour kept its accelerating nature when compared to unknown tumours (p = 0.002).

**Figure 6:**
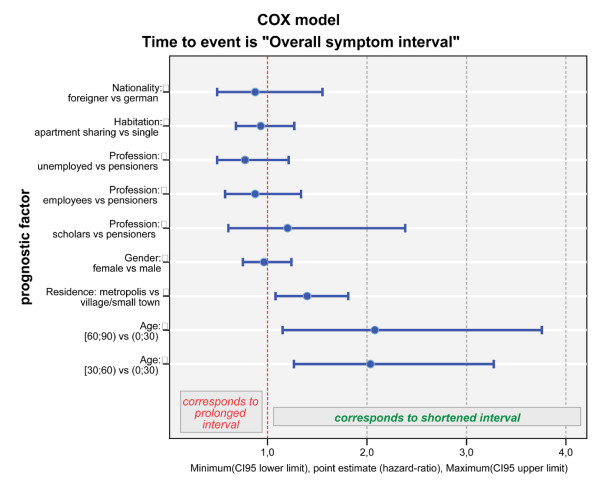
**Cox model of symptom interval**. Step 1 of the Cox model for symptom interval is shown. In this model the factor age <30 years (p < 0.001) and residence in a rural area (p = 0.012) had a significant impact on prolongation of the symptom interval.

To verify the prognostic impact of independent sociodemographic factors on patient delay, professional delay, and symptom interval, we fitted different explorative multivariate Cox regression models for each delay. The criteria differentiation of the tumour and genesis were included into the Cox models, reflecting that for example a known prior tumour might have influenced the patient or the physician to accelerate tumour-specific examinations or diagnostics:

1. A first explorative Cox regression model for **patient delay **starting with the covariate set (p-values of Wald's statistic) profession (p = 0.540), place of residence (p = 0.427), gender (p = 0.123), age at first symptoms (p = 0.418), kind of habitation (p = 0.909), nationality (p = 0.625), differentiation of the tumour (p = 0.423) and genesis (p < 0.0001) yielded the following final result when using a stepwise elimination algorithm: the first factor modelling a statistically significant impact on the length of patient delay was genesis. Information about a prior tumour was associated with a significantly shorter patient delay as compared to when this information was missing, i.e. in case of metastases and unknown primary tumour or primary bone tumour (HR = 3.122 (95%-CI: 2.350–4.146), p < 0.0001). Beside this, only gender remained as a statistically independent covariate in the final model: female gender being associated with a prolonged patient delay as compared to male gender (HR = 0.798 (0.625–1.019), p = 0.072). No significant effect was seen (p-values at step the covariate was eliminated) for age at first symptoms (p = 0.288), the differentiation of the tumour (p = 0.456), profession (p = 0.560), place of residence (p = 0.563), kind of habitation (p = 0.909) and nationality (p = 0.600). The preliminary step 4 of this Cox Model is shown in Figure 4.

2. An explorative Cox regression model for **professional delay **started with the same set of covariates (p-values of Wald's statistic): profession (p = 0.280), place of residence (p = 0.097), gender (p = 0.428), age at first symptoms (p = 0.110), kind of habitation (p = 0.531), nationality (p = 0.501), differentiation of the tumour (p = 0.289) and genesis (p = 0.003). The backward elimination algorithm stopped with modelling a significant impact of the patient's age (p = 0.007) on professional delay: both levels, age between 30 and 60 (HR= 1.379 (95%-CI: 0.957–1.988), p = 0.084) and age over 60 (HR = 1.804 (1.236–2.633), p = 0.002) corresponded to a shortened professional delay when compared to the level age under 30 years. Similarly, the anamnestic status known prior tumour was significantly associated with a shorter professional delay when compared to prior tumour unknown (HR = 1.823 (1.367–2.431), p < 0.0001). The criterion rural habitation corresponded to a prolongated professional delay, but marginal missed level of significance (p = 0.097). Figure 5 resumes the prognostic factors on the professional delay which were still included in the preliminary step 4 of our above described final Cox model.

3. A third explorative Cox regression model was fitted for **symptom interval**, starting with covariate set (p-values of Wald's statistics) profession (p = 0.335), place of residence (p = 0.097), gender (p = 0.989), age at first symptoms (p = 0.103), kind of habitation (p = 0.743), nationality (p = 0.797), differentiation of the tumour (p = 0.165) and genesis (p < 0.0001), and then performing backward elimination. Similar to the above-mentioned professional delay, the factor age has been found to have significant prognostic impact (p = 0.010) on the length of the symptom interval: again both levels of this covariate, age between 30 and 60 years (HR = 1.384 (95%-CI: 0.972–1.970), p = 0.072) and age over 60 years (HR = 1.740(1.209–2.505), p = 0.003) significantly corresponded to a shortened symptom interval when compared to the level age under 30 years. Furthermore, the criterion known prior tumour was significantly associated with a shortened symptom interval as compared to prior tumour unknown (HR = 2.566; 95%-CI 1.915–3.438; p < 0.0001). Subsequently we fitted a Cox model including all sociodemographic factors but excluding the differentiation of the tumour and genesis by using stepwise elimination of covariates. The final model showed again a strong association of higher age with a shortening of the delay (p = 0.001) when comparing the two higher age groups older than 60 years (HR = 1.740 (95%-CI: 1.238–2.445), p = 0.001) and age between 30 and 60 years (HR = 1.992 (1.397–2.841), p = 0.0001) to patients younger than 30 years in our sample. The criterion residence in a metropolis shortened the duration of the symptom interval significantly as compared to residence in villages/small towns (HR = 1.388 (1.075–1.791), p = 0.012). No significant prognostic impact on symptom interval could be detected (p-values shown for step the covariate was eliminated) for profession (p = 0.426), gender (p = 0.789), kind of habitation (p = 0.669) and nationality (p = 0.555) (Figure 6).

To clarify whether the negative impact of a younger age on professional delay and symptom interval could be caused by genesis or differentiation of the tumour, we investigated the influence of the factor age within each of the following groups: prior tumour known, prior tumour unknown, malignant tumour, benign tumour. The factor age had a significant prolonging effect on professional delay and symptom interval in each group.

## Discussion

Our study confirmed that delays in diagnosis of bone tumours are common. The long duration of the symptom interval was caused by both, patient and doctor. These delays are broadly known and identified as a huge obstacle for prompt diagnosis and treatment of bone tumour patients [2-10].

There are several reasons for the long delay. The most important reason is that bone tumours are rare and that symptoms of bone tumours are often vague and misleading [1,2]. Therefore, patient and physician often assume more frequent diseases like tendinitis, sciatic pain or osteoarthritis to be the cause of these symptoms [10].

Aim of this study was to identify the endangered patient for a prolonged patient delay, professional delay or symptom interval by means of sociodemographic risk factors. Therefore we based our statistical exploration on observations of patients with bone tumours, differing with respect to the differentiation of the tumour, prognosis, and necessity of treatment. Only bone tumours with the necessity of a surgical diagnostic or therapeutic intervention have been included into the study. Patients with a radiological clear benign tumour without a necessity of biopsy or treatment have not been included into our study. Therefore we focused only on delays of tumour patients in which early diagnosis or treatment may be essential.

To deal with the problem of detecting histology independent risk factors for a treatment delay in bone tumours we fitted multivariate Cox models including primarily sociodemographic factors and adding genesis and differentiation of the tumour in contrast [21].

It has been purpose but at the same time limitation of our study that the variety of different tumours have not been classified by individual tumour diagnosis however in categories of tumour differentiation and genesis. Hence potential specific characteristics of single tumour types may not be recognized. Furthermore some predictive and prognostic factors may cease to be significant if breaking the cohort down by single tumour types.

In our study we identified certain risk factors leading to a lagged diagnosis of bone tumours and therefore a treatment delay. The prognostic values of the sociodemographic factors are discussed below:

### Age

Our multivariate analyses showed no statistical influence of the factor age on the patient delay. But in contrast the physician needed significantly more time for patients younger than 30 years in comparison to older patients before initiating the first specific therapy: in our multivariate analysis the factor age under 30 years had a significant negative impact on professional delay, i.e. youngest patients showed longer periods until a first specific therapy was realised than older ones.

Consequently, turning the attention on the symptom interval, age under 30 years was associated with longer periods from first symptoms to first specific therapy than more advanced age. Knowing that the age of the patient did not cause a striking impact on patient delay but on professional delay, this delay seems to be overwhelmingly caused by the physician. To ensure that this result had no statistical effect created by the biology of the tumour or a known prior tumour in the sense that malignant tumours occur more frequently in older age and younger patients might have more benign tumours, we included differentiation and genesis of the tumour into the multivariate Cox models: surprisingly, differentiation of the tumour had no significant effect on patients delay, professional delay or symptom interval. If analysing subgroups of patients with malignant or benign tumours in view of the factor age, the criterion age under 30 years maintained the negative impact independently of the differentiation of the tumour. In the same way the prolonging effect of age under 30 years remained independently of tumours genesis: analysing the effect of the criterion age within the subgroups prior tumour known or prior tumour unknown the factor age under 30 years significantly predisposes to a longer symptom interval. Summing up in our study the factor age under 30 years significantly predisposes to a prolonged professional and symptom interval irrespective of tumours differentiation or genesis.

To our knowledge, there are just four studies which investigated the effect of age on delay for bone tumours: three of them included exclusively children or patients younger than 30 years and found a significant influence of the age on symptom intervals [6,10,19]. Another study including only high grade osteosarcoma found no coherence between symptom interval and age [3]. Our study could provide evidence about a prolonged professional delay and symptom interval for young patients with bone tumours. The fact that younger people have longer professional delays and symptom intervals is known for numerous other tumour types [18].

The finding that physicians of our population needed more time to diagnose bone tumours in young people might originate in the rarity of tumours in younger people. Thus it is more likely to go unnoticed by both patients and their health professionals. The diagnosis tumour is frequently not being something the physicians initially consider because the patient is apparently fit, healthy or too young. This assumption is supported by others reporting a high percentage of false diagnosis on patients younger than 30 years with bone tumours, such as 31% of diagnosed tendinitis or 12% of diagnosed uncertain pain at an unseen osteosarcoma [10]. However, ongoing studies of different populations are necessary to verify the histology independent risk factor age under 30 years for a treatment delay of bone tumours.

### Place of Residence

We detected that the criterion rural habitation caused a significant prolongation of the overall symptom interval. Surprisingly, this factor had no significant influence on patient delay but was associated with an extended professional delay. A clue for an explanation of the prolonged professional delay and symptom interval for patients with bone tumours who live in a rural area may be provided by the following facts:

The level of urbanization has long been recognized to affect patient access to health care [22]. It is commonly known that there is a lower supply of physicians practicing in rural areas relative to the population size [23]. In view of the rural health system, Koil et al. have shown that rural practice physicians experience stronger barriers to referral [22]. Another important point might be that rural doctors have less access to continuing education courses, fellow practitioners, and tumour conferences [22,24], all of which serve to educate physicians about bone cancer management. As mentioned above, bone tumours are very rare and therefore a general practitioner will see only few of them in his whole working life [10].

Differences in specialty mix between clinical practise locations may also contribute to differences in referral practice. Rural regions are known to have a greater proportion of general physicians. In contrast, approximately 90% of specialist physicians are located in urban and suburban areas.

Another important fact might be that within our health care system patients from rural regions contact a general practitioner as fast as patients from a metropolis do, but they have a significantly lower rate of contacting specialists such as orthopaedic surgeons or cancer specialists [25]. Other studies concerning bone tumours have shown that the professional delay and therefore the overall symptom interval was significantly extended when patients contacted a general practitioner first in comparison to accident or emergency departments, influenced by a later x-ray examination [6].

In conclusion, we hypothesize that the detected prolonged professional delay and symptom interval of rural patients with bone tumours might be caused firstly by the lower density of physicians and notably bone tumour specialists in rural areas and secondly by the lower further training of rural physicians in diagnosis of bone tumours. So far our database did not differentiate between general physicians and specialists but it is part of our ongoing studies.

### Gender

An extension of patient delay for female gender was found, and the multivariate analyses indicated a negative impact for the female gender concerning patient delay. The gender had neither a statistically relevant effect on professional delay nor on symptom interval. The fact that women with a tumour disease have a prolonged patient delay is ascertained by other workgroups too and was explained by the hypothesis that women more often cited competing priorities of work and family over their own health [18,26-28].

The longer latency of women with bone tumours before first consultation might be due to the different way in which men and women recognise abnormalities, attribute body changes to illness and assess the seriousness of their condition [29].

Concerning bone tumours and patient delay, analogously to our study a longer patient delay for women has been found for osteosarcoma [10]. In contrast to this others found a shortening of symptom interval for women with Ewing's sarcoma [19] and no association between gender and symptom interval for high-grade osteosarcoma [3].

Finally, the question has to be discussed whether a delay of diagnosis and treatment causes a worse patient survival or a reduced quality of life. Trying to challenge the correlation between treatment delay and outcome for the patient, different workgroups stated that a longer treatment delay does not cause a lower survival rate [2,3,6,30]. The lucid explanation of the authors was that a highly malignant and maybe metastasised tumour caused earlier complaints and was therefore correlated to a shorter delay in treatment. But the nature of this statement is exclusively descriptive: the approach to shorten diagnostic- and treatment delay would enable us to detect bone tumours earlier and therefore with a smaller local size or a more localized stage. It is commonly known that a larger tumour is technically more difficult to resect [15,16] and that an earlier date of an operation might augment the opportunity for a successful limb salvage resection [2]. Furthermore, the increasing role of new chemotherapy regiments and bisphosphonate therapy require a diagnosis as early as possible for the best possible success [11,12]. Otherwise some particular tumours may not benefit by shortening diagnosis and treatment due to the fatal biological characteristics. Further on it remains undisputed that the majority of benign bone tumours do not require a treatment at all.

In summary shortening of diagnostic and treatment delay of bone tumour patients is an attractive approach aiming to improve survival and quality of life even if some patients may not benefit by early treatment.

## Conclusion

Early diagnosis and prompt treatment are required for successful management of most bone tumour patients. In our population we succeeded in identifying the histology independent risk factors age under 30 years and rural habitation for a treatment delay in bone tumour patients. These findings of our study should be verified by ongoing studies.

Knowing about the existence of the risk groups age under 30 years and female gender could help the physician to diagnose bone tumours earlier.

The causes for the treatment delay of patients living in a rural area have to be investigated further. If the delay initiates in the lower education of rural general physicians, further training about bone tumours might advance early detection. Hence the outcome of patients with bone tumours could be improved.

## Competing interests

The author(s) declare that they have no competing interests.

## Authors' contributions

CS, MP, HS, KSD, JWPM and PE were all involved in designing the study. CS, MP, JWPM, KSD and PE collected the raw data. HS and CS performed the statistical analysis. CS wrote the draft of the manuscript to which all authors subsequently contributed. All authors made contribution to statistical analyses and interpretation of results. All authors revised the manuscript for important intellectual content and approved the final manuscript.

## Pre-publication history

The pre-publication history for this paper can be accessed here:


